# Lung cancer screening: from imaging to biomarker

**DOI:** 10.1186/2050-7771-1-4

**Published:** 2013-01-16

**Authors:** Dong Xiang, Bicheng Zhang, Donald Doll, Kui Shen, Goetz Kloecker, Carl Freter

**Affiliations:** 1 Division of Hematology and Medical Oncology, Ellis Fischel Cancer Center, University of Missouri, Columbia, MO, USA; 2 Department of Oncology, Wuhan General Hospital of Guangzhou Command, People’s Liberation Army, Wuhan, Hubei, China; 3 Magee-Wowens Research Institute, University of Pittsburgh, Pittsburgh, PA, USA; 4 Division of Hematology and Medical Oncology, Department of Medicine, James Graham Brown Cancer Center, University of Louisville, Louisville, KY, USA

## Abstract

Despite several decades of intensive effort to improve the imaging techniques for lung cancer diagnosis and treatment, primary lung cancer is still the number one cause of cancer death in the United States and worldwide. The major causes of this high mortality rate are distant metastasis evident at diagnosis and ineffective treatment for locally advanced disease. Indeed, approximately forty percent of newly diagnosed lung cancer patients have distant metastasis. Currently, the only potential curative therapy is surgical resection of early stage lung cancer. Therefore, early detection of lung cancer could potentially increase the chance of cure by surgery and underlines the importance of screening and detection of lung cancer. In the past fifty years, screening of lung cancer by chest X-Ray (CXR), sputum cytology, computed tomography (CT), fluorescence endoscopy and low-dose spiral CT (LDCT) has not improved survival except for the recent report in 2010 by the National Lung Screening Trial (NLST), which showed a 20 percent mortality reduction in high risk participants screened with LDCT compared to those screened with CXRs. Furthermore, serum biomarkers for detection of lung cancer using free circulating DNA and RNA, exosomal microRNA, circulating tumor cells and various lung cancer specific antigens have been studied extensively and novel screening methods are being developed with encouraging results. The history of lung cancer screening trials using CXR, sputum cytology and LDCT, as well as results of trials involving various serum biomarkers, are reviewed herein.

## Introduction

Cancer is the leading cause of death worldwide and accounted for 7.6 million deaths in 2008 with primary lung cancer responsible for 1.37 million deaths alone [1]. There are an estimated 226,160 new cases of lung cancer in 2012 in the United States with approximately 160,340 deaths [2]. The lack of sensitive screening tests for early detection of lung cancer plus ineffective treatment for locally advanced and metastatic disease is responsible for the high mortality rate and the dismal overall five year survival rate [3,4]. With the extensive effort for tobacco awareness education, advancements of imaging and combined treatment modalities, the 5-year survival rate of lung cancer has improved marginally from 12% in 1977 to 16% in 2007 [2]. However, if lung cancer is detected at an early stage, complete resection of the tumor may produce a 5-year survival approaching 67% [5]. Therefore early detection of lung cancer by sensitive screening tests could be an important strategy to improve the prognosis of lung cancer.

**Table 1:** LDCT in lung cancer screening

**Trial Name**	**Study design**	**No**. **recruited**	**Characteristics of participants**			**Exsmoker quit (yrs)**	**Year started**	**Report date**	**LC baseline rate (LDCT)**	**Stage I Cancer at Baseline**
			**Age**	**Sex**	**Smoker(PkYr)**					
**NLST **[47]	**LDCT vs CXR**	**53454**	**55–74**	**M/F**	**>=30**	**<15**	**2002**	**2011**	**1.0%**	**63.0%**
**NELSON **[42,95]	**LDCT vs UC**	**15822**	**50–75**	**M/F**	**15–18.75**	**<10**	**2003**	**2016**	**0.9%**	**63.9%**
**ITALUNG **[39]	**LDCT vs UC**	**3206**	**55–69**	**M/F**	**>=20**	**<10**	**2004**	**NR**	**1.5%**	**47.6%**
**DEPISCAN **[38]	**LDCT vs CXR**	**765**	**50–75**	**M/F**	**>=15**	**<15**	**2002**	**2006**	**2.38%**	**0.9%**
**DANTE **[37,96]	**LDCT vs UC**	**2472**	**60–74**	**M**	**>=20**	**<10**	**2001**	**2007**	**2.2%**	**57%**
**DLCST **[97]	**LDCT vs UC**	**4104**	**50–70**	**M/F**	**>=20**	**<10**	**2004**	**2016**	**0.8%**	**58.8%**
**LSS **[34,36]	**LDCT vs CXR**	**3318**	**55–77**	**M/F**	**>=30**	**<10**	**2000**	**2005**	**1.9%**	**53.3%**

LDCT= low-dose computed tomography; CXR=chest X-ray; NC=usual care; M=male; F=female; PkYr=packs/year; yrs=years; f/u=follow-up; NR=not reported; LC=lung cancer.

The results of primary lung cancer screening trials using imaging modalities such as chest X-RAYs combined with sputum sampling, CT scans and LDCT will be reviewed. In addition, the role of serum biomarkers and biological modalities, circulating DNA and RNA, exosomal microRNA and circulating tumor cells (CTCs) and thermogram and nanopore sensor technologies will be discussed.

## History of lung cancer screening with chest X-ray

The first large prospective trial using chest X-Ray as a screening tool for the early detection of lung cancer was performed in the UK and published in 1968. In that study there were 29723 men aged 40 years or older who had six-monthly chest radiograph screening for three years compared with a control group of 25311 men who received chest radiograph at the beginning and the end of the study. Lung cancer annual mortality rate did not differ statistically in the two groups, with 62 deaths in the screened group and 59 in the control group, but the 5-year survival rate for participants with lung cancer diagnosed by screening chest radiographs was 23% versus 6% in the control group [6,7].

## Chest X-ray and sputum cytology

There are four randomized trials integrating CXR with sputum cytology. The first three trials were sponsored in the United States by the National Cancer Institute (NCI): the Mayo Lung Project (MLP), the Johns Hopkins Lung Project (JHLP), and the Memorial Sloan-Kettering Lung Project (MSKLP) [8-10].

The MLP randomized 9211 participants from 10,933 high risk men aged 45 years or older to CXR and sputum cytology annually as the control group versus CXR and sputum cytology every 4 months in the screening group for 6 years. The study reported that there were 206 cases of lung cancer diagnosed in the screening group and 160 cases in the control group, with significantly earlier stages of lung cancer diagnosed in the screening group and an improved 5-year survival. However, the trial did not show any statistical disease-specific mortality difference between the two study groups from lung cancer with follow-up over 20 years [8,11,12].

The MSKLP and the JHLP trials randomized participants aged 45 or older to annual CXR with (screening group) or without (control group) sputum cytology analysis every 4 months. The MSKLP study enrolled a total of 10,040 subjects and diagnosed 144 lung cancer cases in both groups but there was no difference in stage distribution, overall survival or disease specific mortality between the two groups [10,13]. The JHLP study randomized 10,386 participants and noted 194 cases of lung cancer in the screening group compared to 202 cases in the control group. Akin to the MSKLP trial, the final results of JHLP showed no differences in overall survival or disease-specific mortality between the study groups [14-16].

A Czechoslovakian trial randomized 6364 high-risk participants aged 40 or older to CXR and sputum cytology every 6 months for 3 years as the screening group versus CXR and sputum cytology at the beginning and the end of 3 years in the control group [17]. There were 39 cases of lung cancer in the screening group versus 27 cases in the control group. However, there was no difference in disease-specific mortality between the two groups at follow-up of 20 plus years [18].

From the aforementioned results of five randomized trials, screening with CXR with or without sputum cytology failed to reduce disease-specific mortality from lung cancer in high risk populations. Nonetheless, in 1993 another large multicenter NCI-sponsored clinic trial, the Prostate, Lung, Colorectal and Ovarian (PLCO) Cancer Screening Trial, randomized 154,901 individuals aged 55 to 74 to receive annual CXR for 3 years or standard medical care as controls. The screening ended in 2006 and the final results showed that there were 1213 lung cancer specific deaths in the screening group versus 1230 in the control population. There was no difference in terms of stage and histology [19].

## CT scan and lung cancer screening

Due to the high cost and false-positive results causing over-diagnosis and treatment, CT scanning was not initially considered to be a useful screening test for cancer detection. However, advances in imaging technology and the development of fast LDCT have renewed interest in CT scanning as a potential effective screening test for early detection of lung cancer. Pilot studies using LDCT conducted in New York [20,21] and Japan [22] for lung cancer screening reported that stage I lung cancer was detected in 80-90% of newly diagnosed cases. Such encouraging results warrant further clinic trials using LDCT as a screening tool for lung cancer detection in high risk individuals.

## LDCT and lung cancer screening

There have been several uncontrolled LDCT screening studies for lung cancer since 1992 (Table 1). The first study was conducted in Japan and compared LDCT with CXR for lung cancer screening in a high-risk population [23]. This study enrolled 1369 high-risk individuals to receive CXR and LDCT scans twice a year. The study showed that there were 15 cases of lung cancer detected by LDCT, but only 4 cases of the 15 were detected by CXR. Among the 15 cases of lung cancer, 14 (93%) were stage I and the mean tumor size was 1.6 centimeter [24]. In an update of that study, the five year survival rates for lung cancers detected by initial and repeated LDCT screening were 76.2% and 64.9%, respectively.

The second large uncontrolled study was also done in Japan [25,26] and enrolled 5483 participants aged 40 to 74 to annual LDCT with annual sputum cytology and 3967 participants also had CXR. The results showed that 63 lung cancers were diagnosed and 57 of them were detected by LDCT alone. Fifty one (89.5%) of the 57 cases were stage I. Ten year survival of the 59 lung cancers detected by LDCT screening was estimated at 86.2% [27].

The third non-randomized trial, the New York Early Lung Cancer Action Project (ELCAP) [21], recruited 1000 asymptomatic smoking participants aged 60 or over to evaluate annual LDCT scan as a screening test for lung cancer compared with X-Ray. There were 27 lung cancer cases detected by LDCT but CXR only diagnosed 7 of the 27 lung cancer cases observed by LDCT. Twenty three (85%) of the lung cancers were stage I by LDCT whereas CXR only detected four stage I lung cancer (14.8%).

The fourth study was conducted at the Mayo Clinic and included 1520 participants aged 50 or over with at least a 20 pack-year history of smoking who underwent annual LDCT and sputum cytology for five years. The results showed that 17 of 28 non-small cell lung cancer cases detected were stage I and mean tumor diameter was 14.4 mm [28-30]. However, there was no difference in lung cancer related mortality rates between this study and the Mayo Lung Project in age and sex matched subset analysis.

In 2006, Henschke *et al.,* of ELCAP published the results of an international collaboration (I-ELCAP), in which 31567 high-risk asymptomatic participants aged 40 or over underwent LDCT screening from 1993 to 2005 at intervals of 7 to 18 months [31]. A total of 484 lung cancer cases were diagnosed (405 initial, 74 at follow-up, 5 interval cancers). Among those 484 cases, 412 cases were stage I with an estimated 10 year survival rate of 88% compared to 80% for all lung cancer patients. The survival rate of 302 patients with stage I lung cancer detected by LDCT screening undergoing surgical resection was 92%. The study results were encouraging but the study was criticized because of funding sources from a tobacco company [32]. Other important issues were lead-time bias and over-diagnosis bias due to the slowly progressive nature of stage I lung cancer as well as the seemingly short 40 months median follow-up and less than 20% of them followed for more than 5 years.

In response to the I-ELCAP results, Bach *et al.,*[33] reviewed the results of three uncontrolled LDCT screening trials conducted at Instituto Tumori in Milan,Italy, the Moffitt Cancer Center in Tampa, FL, and the Mayo Clinic, Rochester, MN in which 3246 asymptomatic, high-risk participants underwent at least three annual LDCT screenings and had a median follow-up of 3.9 years. The results showed that 144 lung cancers were detected by LDCT screening compared with 44.5 cases expected, which is more than a three-fold increase in the number of newly-diagnosed lung cancer cases. Among 144 cases, there were 96 (78%) stage I/II non-small cell lung cancers. However, there was no significant decrease in lung cancer related mortality rates, with 38 deaths due to lung cancer in the trials compared with 38.8 deaths predicted. In addition there was no decrease in the number of advanced lung cancers diagnosed with 42 individuals compared to 33.4 predicted.

## Randomized controlled trials using LDCT for lung cancer screening

Compared with non-randomized studies, randomized controlled trials (RCTs) are ideal studies to detect a real benefit of any screening modality in terms of differences in mortality and recurrence of disease by comparing screening group with controlled group and to avoid lead time and length bias and overdiagnosis.

Nine randomized controlled trials evaluating LDCT to screen lung cancer are ongoing worldwide [34-45]. [46] The Lung Screening Study (LSS) was initiated in 2000, whereas the NLST published the first trial results [47] showing a relative 20% reduction in lung cancer specific deaths among participants screened with LDCT versus CXR. The NLST trial in 2002 recruited 53,454 high-risk participants aged 55 or older to undergo three screenings at 1-year intervals with either LDCT or CXR. The LDCT group noted 247 lung cancer deaths per 100,000 person-years compared to 309 deaths in the CXR group. The NLST trial was acclaimed as a major breakthrough in the lung cancer screening field and showed clear evidence of a significant reduction in lung cancer deaths, but raised concerns regarding how to define the high-risk populations and who would benefit from LDCT screening; what is the optimal time to start LDCT screening; how long to follow patients and what intervals to screen; and lastly the overwhelming financial cost of LDCT screening. Time and further research may provide definitive answers regarding the impact of LDCT screening on lung cancer specific mortality at the population level. Additional ongoing randomized trials will hopefully address some of these questions and validate the NLST results.

The National Comprehensive Cancer Network (NCCN) published Lung Cancer Screening Guidelines [48], recommending annual LDCT screening for high-risk individuals aged 55 to 74 who quit smoking less than 15 years with at least a 30 pack-year smoking history. The guidelines suggested algorithms to evaluate lung lesions such as solid nodules of different size and ground glass opacities and at what intervals, but uncertainties concerning the duration of follow up and at what age to stop remains to be addressed. Nonetheless, this is the first lung cancer screening recommendation attempting to reduce lung cancer related mortality.

## Serum biomarkers and screening for lung cancer

Currently, there are no validated biomarkers for early lung cancer detection. Extensive efforts have been made for the development of useful biomarkers and novel biomarkers from recent studies will be discussed here.

## Circulating tumor cells (CTCs)

CTCs are circulating and free floating malignant cells that have migrated from the primary cancer or metastatic sites in the peripheral blood. Advanced techniques have been developed to identify, isolate and characterize these CTCs [49]. Different from traditional invasive procedures like biopsies, CTCs represent a potentially easy source of tumor tissue for diagnosis, characterization and monitoring of solid tumors. CTCs have been evaluated as a surrogate metastatic marker for primary lung cancer [50]. The CTC-chip, an innovative micro-device developed at Harvard, proved feasible to identify and isolate CTCs in the blood of metastatic lung cancer patients [51]. Further studies to confirm the clinical value of CTCs in the early detection, characterization and monitoring of lung cancer are ongoing.

## Free circulating DNA

In 2003, Sozzi *et al.,* published a case–control study demonstrating that the quantity of free circulating DNA can be measured in lung cancer patients by real-time quantitative polymerase chain reaction (RT-PCR) of the human telomerase reverse transcriptase gene (*hTERT*) [52]. Subsequently Sozzi *et al.,* designed a 5-year prospective trial that enrolled 1,035 heavy smokers aged 50 years or older who were monitored by annual LDCT for 5 years and plasma circulating DNA levels were measured by RT-PCR. The authors reported no improvement in the accuracy of lung cancer screening by LDCT in heavy smokers by measurement of the baseline assessment of plasma circulating DNA level, but higher levels of plasma circulating DNA at surgery was a risk factor for aggressiveness of lung cancer with a decreased 5-year survival [53]. They hypothesized that only rapidly growing lung cancers would be able to produce a sufficient amount of plasma circulating DNA to be sensitive as a marker. In addition, others have noted that free circulating DNA measured by human *hTERT* gene is not specific for lung cancer and that the level of free circulating DNA may be influenced by other benign diseases [54].

## Plasma proteomics

Abundant proteins in the plasma are an underutilized source with valuable information for clinical usage [55]. Chairs *et al.,* studied the thermal properties of plasma by highly sensitive differential scanning calorimetry (DSC) which can generate unique and statistically different DSC thermograms in several diseases, such as SLE, Lyme disease, and rheumatoid arthritis or cancers including cervical, lung, melanoma, ovarian, and uterine [56-60]. In Xiang *et al.,* a case–control study [61] involving 20 patients with early stage lung cancer and 100 healthy volunteers, DSC thermograms from patients with lung cancer generated a unique characteristic thermogram that differed statistically from healthy controls and suggested that DSC was sensitive to properties of plasma proteins other than their charge and mass. Such studies hypothesized that characteristic thermogram changes in shape and position in lung cancer can arise from changes in the concentrations of different plasma proteins, including tumor antigens, from cancer-related protein-protein interactions, or cancer-secreted peptides binding to other plasma proteins. In a subsequent study [62] of 30 patients with early stage lung cancer and 109 healthy controls, the authors validated their hypothesis and suggested that early stage lung cancer may have a characteristic signature thermogram profile that may serve as a biomarker for the diagnosis of lung cancer. A large scale clinical study is on-going to further characterize the sensitivity and specificity of this assay.

## Exosomal microRNA

The discovery of tumor-derived exosomes was first reported in the peripheral blood of ovarian cancer patients in 1979 [63,64]. Exosomes are small membrane vesicles containing common proteins generic to all exosomes as well as tumor associated proteins (antigens) unique to their origin [65]. Tumor cells can secrete elevated levels of exomes containing tumor antigens (Her2/Neu and gp100), which can be detected in serum, malignant effusions and ascites [66,67]. Ratajczak *et al.,*[68]. showed that horizontal transfer of exosome associated mRNA may play a key role in signal transduction leading to cell proliferation. Further studies by Valadi *et al.,*[69,70]. reported that both cellular mRNA and microRNA can be carried by exosomes to remote target cells and functional proteins can be identified in target cells. Based on these correlations, Taylor *et al.,*[71]. reported that patients with ovarian cancer demonstrated unique exosomal microRNA patterns which were characteristically different from the pattern from healthy controls, which suggested that cancer specific microRNA within exosomes may be useful as a putative diagnostic biomarker for early detection of cancer.

Based on evidence that specific microRNA profiles are unique to different types of cancers [72], Rabinowits *et al.,*[73]. designed a case–control study and enrolled 27 patients with various stages of lung adenocarcinoma and 9 healthy controls, and evaluated the concentration of exosomes and microRNA. The authors reported that the mean exosome concentration was 4 times higher in the lung adenocarcinoma group (2.85 mg/mL) compared to the control group (0.77 mg/mL). The mean microRNA concentration was 2.3 times higher in the lung adenocarcinoma group (158.6 mg/mL) versus the control group (68.1 mg/mL). A significant difference in exosome and miRNA concentration between lung cancer patients and controls suggest that circulating exosomal microRNA might be a useful biomarker for early detection of lung cancer.

## Nanopore sensor

In the last twenty years, various sensitive quantitative techniques have been developed to detect and analyze macromolecules at a single molecular level in biomedical research [74]. With increased understanding of short tumor-specific microRNAs playing a regulatory role in tumor cell proliferation and differentiation and an association between specific microRNA profiles with different types of cancers, detecting microRNA by utilizing highly sensitive single-molecular assays such as fluorescence correlation spectroscopy [75], nanoparticles and single-molecule fluorescence [74] showed diagnostic potential for clinic application for the early detection of cancer and monitoring response to treatment. In order to avoid complicated chemical labeling with fluorescence dye and target amplification by the aforementioned assays, Wang *et al.,*[76]. described an α-haemolysin protein based nanopore sensor technology to detect microRNAs at the molecular level from blood samples in six lung cancer patients without amplification via microRNAs. The nanopore sensor is capable of detecting single nucleotide difference with *let-7* microRNAs. This strategy may be used in concert with unique lung cancer microRNA profiles for lung cancer screening and/or monitoring treatment responses in lung cancer patients.

## Telomerase

Telomerase, a ribonucleoprotein complex with reverse transcriptase activity to synthesis DNA termini at the end of eukaryotic chromosomes during each cell division, plays a vital role in the replication of chromosomal termini or telomeres. It is inactivated in most normal tissues at an undetectable level and reactivated or upregulated in all cancer tissues investigated, such as breast, prostate, lung, liver, and pancreatic cancer [77-79]. Compared to other cancer markers, the level of telomerase activity may be increased gradually from very early tumorigenesis and can be detectable even before cancer patients experience any symptoms [80], which suggests that telomerase activity maybe a useful biomarker for the early detection of cancer.

Miyazu *et al.,*[81]. evaluated telomerase protein expression in normal bronchial epithelia among 206 high-risk lung cancer individuals in which bronchoscopy was performed every 3 to 6 months. Fifteen lesions were detected in 12 patients. Human telomerase reverse transcriptase was measured and 11 of the 15 lesions that were telomerase negative did not develop lung cancer after photodynamic therapy whereas 4 of the 15 lesions that were positive for telomerase progressed to lung cancer. Such findings suggest that telomerase activity may be a useful biomarker for detection of lung cancer whose telomerase activity is upregulated in early stages of cancers or pre-cancerous cells. Further well-designed clinical studies are needed to help elucidate the role of telomerase in tumorigenesis and validate the role of telomerase as a biomarker.

## Circulating RNA

Aberrant mRNA expression from normal genes has also been investigated in association with tumorigenesis. Using reverse transcription polymerase chain reaction (RT-PCR), studies have demonstrated the detection of tyrosinase mRNA in whole blood samples of melanoma patients [82] and telomerase RNA in the serum from breast cancer patients [83]. These results prompted investigators to explore the use of circulating RNA as a biomarker for detection of lung cancer. The translational production of circulating 5T4 mRNA is a trophoblast glycoprotein present in epithelial malignancies and is a potential biomarker for cancer detection. Kopreski *et al.,*[84]. evaluated serum for 5T4 mRNA in 5 patients with breast cancer, 14 patients with non-small-cell lung cancer and 25 normal controls. The circulating 5T4 mRNA was detected in 42% of cancer patients (8/19) and 12% of normal controls (3/25). These results demonstrate that circulating mRNA may be a biomarker for early lung cancer diagnosis.

## Miscellaneous biomarkers

There are several other molecular candidates that have been used as biomarkers. The LunX (lung-specific X protein) is a human lung-specific gene identified in 2001 [85]. Mitas *et al.,* reported that LunX mRNA was overexpressed in 14 of 24 (58%) blood samples from NSCLC patients [86]. Cytokeratin 19 is an acidic subunit expressed in carcinoma cells as well as on normal epithelia whereas CYFRA21-1 is a cytokeratin 19 fragment in the sera of patients with lung cancers. CYFRA21-1 has been studied extensively and one study recruited 104 patients with malignancies and 42 healthy controls and showed that CYFRA21-1 was overexpressed in 86% of malignant cases, especially in lung cancer (9 of 16 cases) [87].

Using fluorescent microsatellite analysis, Beau-Faller *et al.,* evaluated DNA microsatellite in 34 patients with lung cancer, including 11 patients with small cell lung cancer (SCLC) and 23 patients with NSCLC and 20 healthy controls. A panel of 12 microsatellites was selected to perform allelotyping. Plasma DNA allelic imbalance (AI) was noted in 88% of cancer patients (30/34) compared to no plasma or bronchial DNA alteration in 20 healthy controls [88].

## Discussion

Decades of research have evaluated various approaches for lung cancer screening, including routine chest radiograph, sputum cytology, CT scanning, LDCT and, most recently, molecular biomarkers. Early screening tests of high risk individuals based on sputum cytology or chest X-Ray have not shown improvement in disease-specific survival. Chest LDCT scan has been proven as an effective tool for the detection of early stage resectable disease. Indeed, results from the NLSCT showed a 20 percent mortality reduction in high-risk participants screened with LDCT compared to those with chest X-Rays. Recent NCCN guidelines endorse LDCT as a screening tool for lung cancer. However, questions remain regarding the definition of high risk patients; how long and how frequent to screen; and how to address the false positive results and high cost and potential toxicity from radiation exposure. Additional randomized trials using LDCT are ongoing and will address these unanswered questions and may eventually demonstrate the real impact of early detection on mortality at a population level.

Several serum tumor protein markers have been studied extensively, and are currently available for blood testing, such as carcinoembryonic antigen, serum cytokeratin 19 fragment, and progastrin-releasing peptide, but none of them is satisfactory for diagnosis of early stage lung cancer because of their relatively low sensitivity and specificity in detecting the presence of cancer cells [89-91]. Different and innovative approaches must be explored to tap the wealth of serum tumor protein pools like DSC thermogram profiling for earlier detection of lung cancer, which appears promising according to case control study results.

With the advances of our understanding of key cellular changes of lung cancer and the rapid development of new technologies, recent diagnostic approaches have focused on genomics and proteomics. The Human Genome Project (HGP) announced the first working draft in 2000 and opened the genomic era, which has provided opportunities for gene expression analysis and several studies have identified differences in gene expression between normal and lung cancer cells using techniques such as serial analysis of gene expression [92] and cDNA microarray [93,94].

Researchers have been studying tumorigenesis, targeting subtle changes at the molecular level to facilitate early diagnosis, identify clinically relevant biological triggers related to cancer development, and identify useful biomarkers. In this regard, CTCs, exosomal microRNA, free circulating DNA, telomerase, LunX and CYFRA21-1 have demonstrated encouraging results. Investigators have taken advantage of technical advances that allow rapid high-throughput assays to interrogate the genome, proteome, and epigenome. Results of such Phase I studies are expected to be reported in 2012.

Based on screening guidelines suggested by the NCCN, significant strides have been made in effective screening methods to diagnosis lung cancer at early stages for high risk patients with a 30 pack-year smoking history. But challenges in early detection of lung cancer remain. As one can anticipate, in the near future, effective biomarkers or evidence-based image scans used alone or in combination may provide the clinician with effective tools for the early detection of lung cancer in the era of molecular and digital oncology, and hopefully alter the natural course of lung cancer and improve the survival of patients with lung cancer.

## Competing interest

The authors declare that they have no competing interests.

## Authors’ contributions

All authors participated equally in draft writing and revising the manuscript. All authors read and approve the final manuscript.
